# Niche Dynamics of Alien Plant Species in Mediterranean Europe

**DOI:** 10.1111/gcb.70379

**Published:** 2025-08-01

**Authors:** Luigi Cao Pinna, Laure Gallien, Tommaso Jucker, Milan Chytrý, Greta La Bella, Alicia T. R. Acosta, Marta Carboni

**Affiliations:** ^1^ Department of Science Roma Tre University Rome Italy; ^2^ School of Mathematics and Statistics University of Glasgow Glasgow UK; ^3^ University of Grenoble Alpes, CNRS, University Savoie Mont Blanc LECA Grenoble France; ^4^ School of Biological Sciences University of Bristol Bristol UK; ^5^ Department of Botany and Zoology Faculty of Science, Masaryk University Brno Czechia

**Keywords:** acclimatization, invasion success, invasive species, niche dynamics, niche filling, phenotypic plasticity, rapid adaptation, species traits

## Abstract

Humans have spread plants globally for millennia, inadvertently causing ecological disruptions. Apart from their negative effects, biological invasions provide a unique opportunity to study how species modify their niche when confronted with novel environments. Focusing on the Mediterranean Basin, we assessed (1) which traits influence niche dynamics, and (2) whether niche conservatism or niche shift promotes invasion success. We selected the 80 most widespread alien vascular plant species in Mediterranean Europe and compiled data on their distributions in their native and invaded ranges. We then tested how a species' residence time, biogeographic origin, dispersal ability, functional traits, and intraspecific trait variability (ITV) influence its niche dynamics following invasion. Using already published independent data, we finally assessed whether niche dynamics can explain different dimensions of invasion success (quantified as regional spread or local abundance). We found that niche shifts were common (71% of species) and were mostly driven by species failing to occupy all suitable environments in their invaded range (unfilling), regardless of residence time. Niche unfilling and niche expansion were more important in species with high intraspecific trait variability introduced from non‐Mediterranean biomes (temperate or tropical). Niche expansion was also greater in species with long‐distance dispersal, a narrow native niche, and bigger seeds. Interestingly, invasion success correlated more with a species' ability to conserve its niche and residence time than with niche expansion. Niche shifts were better predicted by species traits than residence time. For example, high adaptive and acclimatization potential (inferred from high intraspecific trait variability) favored niche shifts in general, and long‐distance dispersal favored niche expansion. Understanding how these traits relate to niche dynamics is important since a species' ability to conserve and fill its niche is, in turn, a good predictor of invasion success.

## Graphical abstract

In this study, we analyzed some of the most widespread alien plant species in Mediterranean Europe to understand how functional traits and species characteristics influence niche shifts, and how these, in turn, affect invasion success. We found that a species' biogeographic origin, dispersal distance, functional traits, and intraspecific trait variability were strong predictors of niche dynamics. Notably, species with greater intraspecific trait variability shifted their niche the most, but invasion success was favored by niche conservatism. Understanding how traits and their variability influence niche dynamics is important and could help predict species' invasion success.

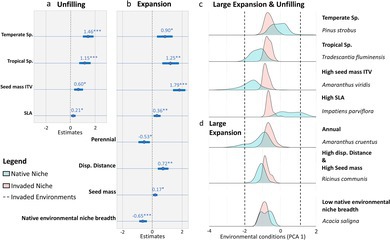

## 1 Introduction

People have been moving plants around for millennia, often with unexpected and ecologically disastrous consequences (Pyšek et al. 2020; van Kleunen et al. 2015). But this process of reshuffling floras also presents an opportunity to study niche dynamics, the process through which species modify their niche when confronted with a novel ecological setting (Atwater et al. 2018; Colautti and Lau 2015). A central tenet of niche theory has been that species largely conserve their niches on short (“ecological”) timeframes (i.e., niche conservatism, Wiens et al. 2010) and tend to occupy similar environments when introduced into new areas (Petitpierre et al. 2012; Strubbe et al. 2013). However, many recent invasion studies have challenged this view, reporting cases of alien species that have considerably shifted their realized niche following invasion (Atwater and Barney 2021; Atwater et al. 2018; Broennimann et al. 2012; Early and Sax 2014). Studies examining these rapid niche shifts have yielded contrasting results, with some studies showing niche shifts are common (Dellinger et al. 2016; Liu et al. 2020) and others concluding that shifts are rare (Liu et al. 2020; Petitpierre et al. 2012; Sychrová et al. 2022). These inconsistencies are partly attributable to methodological differences across studies (Bates and Bertelsmeier 2021; Guisan et al. 2014), but also to the dependence of niche shifts on the ecological characteristics of the species itself (e.g., Dellinger et al. 2016; Li et al. 2014; MacLean and Beissinger 2017). Identifying which species are most likely to shift their realized niche is particularly relevant if this ability can contribute to the invasion success in new environments (Sotka et al. 2018; van Kleunen et al. 2010). However, the species' ecological characteristics favour niche shifting, and the link between niche dynamics and invasion success is poorly understood.

Niche dynamics, resulting in either niche conservatism or niche shifts, can be assessed by comparing species' niches based on environmental data and occurrence records in their native and invaded ranges (Di Cola et al. 2017). The most commonly used framework to quantify and statistically test for niche shifts in environmental space generally restricts this comparison to the shared, analogue environmental conditions between the native and invaded ranges (Guisan et al. 2014). This is because the colonization of environments not available in the native range is by some considered more challenging to interpret. According to this framework, niche shifts in the analogue climates can be ascribed to releases from biological or geographical constraints (realized niche shifts), as well as to processes of adaptation (fundamental niche shifts). Specifically, when colonizing a new region, invasive species can either maintain, expand, or fail to fill their native niche (Pearman et al. 2008). The realized niche quantified in the invaded range can thus be divided into three parts: (1) niche stability, the part of the niche that the species occupies in both its native and invaded ranges; (2) niche expansion, the portion of environmental space occupied exclusively in the invaded range; and (3) niche unfilling, the set of environmental conditions occupied only in its native range.

Niche unfilling may be a consequence of dispersal limitation in the invaded range (e.g., dispersal barriers or poor dispersal ability; Giulio et al. 2022; Li et al. 2014), but may also occur if positive interactions have been lost during invasion (e.g., if mycorrhiza or other mutualists have not been introduced at the same time; Grove et al. 2017; Menzel et al. 2017). Conversely, niche expansion may occur if dispersal barriers were limiting the native range of a species (Bates and Bertelsmeier 2021), if enemy release and new facilitations occur in the new range (Keane and Crawley 2002) but also through species acclimatization to new climatic conditions and other abiotic factors (Bujan et al. 2021; Lancaster et al. 2015) and/or if species evolve novel local adaptations that allow them to spread outside the limits of the environmental conditions occupied at home (Gallien et al. 2016; Colautti and Barrett 2013). In sum, dispersal limitation, biotic interactions, and potentially local acclimatization or adaptations are the main processes that are likely to influence niche dynamics in the invaded range, and species ecological characteristics related to these processes may help explain variability across taxa.

Dispersal limitations play a major role in the spatial patterns of invasive species. These limitations are the result of a combination of factors, including dispersal abilities and species‐specific introduction history. Introduction history is particularly relevant as it relates to residence time, propagule pressure, and the spatial patterns of introduction. However, it also relates to the likelihood of overcoming landscape barriers in the invaded range. Species with the intrinsic ability to disperse over great distances are less likely to have been dispersal limited at home (and thus less likely to expand their niche after introduction) but also in the invaded range (i.e., more likely to rapidly fill their niche once introduced). Recently introduced species with poor dispersal ability may thus not yet have reached all the environmental conditions they occupy in their native range (stage of unfilling). In contrast, species introduced longer ago with good dispersal ability will have had more time to overcome barriers and fill or potentially expand their niche (Li et al. 2014). Consequently, both residence time and species dispersal ability could influence niche unfilling and expansion (Menuz et al. 2015).

Biotic interactions are also well known to be crucial in the invasion success of introduced species. Enemy release in the invaded range (i.e., the loss of predators, parasites and competitors, Brian and Catford 2023; Keane and Crawley 2002) can lead to an expansion of the realized niche in new environments free of native antagonists. Species with traits typically associated with slow resource acquisition, such as short stature, slow growth, and big seeds (e.g., Gioria et al. 2023; Richardson and Pyšek 2006; van Kleunen et al. 2010), are poor competitors in disturbed areas and may be more likely to expand their niche when they are released from their antagonists that otherwise limit their establishment. In contrast, plant species that are highly competitive in resource acquisition at home (e.g., high SLA) might be relatively unaffected by release from competitors, especially in disturbed habitats. However, these fast‐growing plants often have low investment in defences against herbivores (e.g., high SLA often means high palatability, de Vries et al. 2019), so they might instead experience greater release from predators or parasites, resulting in niche expansion and potentially better niche filling (Bates and Bertelsmeier 2021). In addition to plant traits, the environmental context in which a species evolved, like its biome of origin (Mucina 2019; Olson et al. 2001), might also be important for explaining niche dynamics (e.g., if the new range hosts similar mutualists and/or antagonists because it shares the same bioclimatic conditions as the species' native range; Cao Pinna et al. 2021). Similarly, species with broad realized niches at home are less likely to be constrained by dispersal barriers or biotic interactions and are more likely to occupy suitable environments in novel areas with less expansion and less unfilling (Mitchell and Power 2003). These species would not find many additional environments to expand their niche compared to species with narrow environmental tolerances (Bates et al. 2020; Gallien et al. 2019; Early and Sax 2014). Thus, species' ecological characteristics, bioclimatic pre‐adaptations, and native niche breadth can potentially predict expansion and unfilling. However, despite this strong theoretical basis, these effects on niche dynamics have only been explored by a few multi‐taxa studies. Bates et al. (2020) and Li et al. (2014), for example, explained niche shifts based on species' native range size and characteristics. An even smaller number of studies, such as those by Sychrová et al. (2022) and Atwater et al. (2018), have found that biogeographic origin and growth forms significantly influence the niche dynamics of alien plants.

Finally, while changes in dispersal limitations and biotic interactions will mainly affect a species' realized niche, rapid evolution and local adaptation in response to novel conditions may also result in shifts in its fundamental niche (when appropriately proved, see Bates and Bertelsmeier 2021). These processes may play an important role in driving niche dynamics following the invasion, given that several invasive species have been found to have rapidly evolved in their new ranges as a result of increased genetic variation, interspecific hybridization, genetic bottlenecks, or natural selection in novel environments (Bock et al. 2021; Smith et al. 2020; Gallien et al. 2016; Colautti and Barrett 2013). For instance, Dellinger et al. (2016) hypothesized that sexually reproducing plants should be more likely to shift their niches following invasion due to their higher genetic diversity. However, the reproductive system is only one proxy of genetic diversity (Colautti and Lau 2015; Smith et al. 2020), and other characteristics may be associated with a greater ability to adapt (Colautti and Barrett 2013). For example, intraspecific variation in functional traits (ITV) reflects a combination of phenotypic plasticity and heritable genetic variation (Karbstein et al. 2020). Consequently, we might expect that higher ITV should be related to greater evolutionary and adaptive potential (Karbstein et al. 2020) and theoretically also to a greater potential for fundamental niche shifts during invasions. Similarly, higher ITV might also be related to a greater likelihood of acclimatization during invasion, and thus to more realized niche shifts due to plasticity (Bujan et al. 2021; Lancaster et al. 2015). While distinguishing between realized and fundamental niche shifts requires experimental manipulations (Bates and Bertelsmeier 2021), ITV may be an important indicator of both. Yet, the relationship between species' ITV and their niche dynamics remains to be tested.

To test these ideas, we compiled distribution and trait data for the most widespread alien plants in Mediterranean Europe. We focused on the Mediterranean Basin, one of the world's biodiversity hotspots (Mittermeier et al. 2011), because it has a long history of human exploitation that has led to the introduction of numerous alien plant species (Underwood et al. 2009). This makes the Mediterranean Basin an ideal arena to study the niche dynamics of introduced alien plants, as within one biogeographically and climatically defined region, we can compare many species with contrasting introduction histories, dispersal abilities, and functional traits. Using this unique dataset, we quantified niche shifts by comparing alien species' niches in Mediterranean Europe with their niches at home and tested whether these shifts are underlain by expansion and/or unfilling. Then we tested the following overarching questions:

1. Is niche shift common in invaders of Mediterranean Europe?
2. Which ecological characteristics influence niche filling, and which drive niche expansion?
3. Is invasion success in Mediterranean Europe more frequent in niche‐shifting species or in species that conserve their niche?

## 2 Material and Methods

### 2.1 Study Area and Alien Species Records

To quantify niche shifts in plant species introduced in Mediterranean Europe (including Anatolia and Cyprus), we first identified the most widespread alien plants in the study area and then quantified their native and invaded niche using a combination of presence records and data on their bioclimatic envelope. The invaded range included the Mediterranean biogeographic region in Portugal, Spain, France, Monaco, Italy, Malta, Croatia, Bosnia and Herzegovina, Montenegro, Albania, Greece, Turkey, and Cyprus, as defined by the European Environmental Agency (EEA; Cao Pinna et al. 2021). We focused on alien plant species identified by Cao Pinna et al. (2021) by selecting species with at least 30 recorded presences across 130,000 field‐based vegetation plots in the study area. These data from the European Vegetation Archive (EVA; Chytrý et al. 2016) provide a robust estimate of the naturalized flora of an area. From this set of species, we then selected the 100 most widespread neophytes (i.e., species introduced after 1500 ad), as identified by Cao Pinna et al. (2024), and excluded species that are mostly cultivated and rarely found to be naturalized in the wild. These species were naturalized in at least two of the countries of our study area (with a mean of six) according to the GloNAF dataset (van Kleunen et al. 2019, as shown in Data S1). For these neophytes, we extracted the global occurrences from the Global Biodiversity Information Facility (GBIF; accessed in 2020). We excluded GBIF records older than 1950 and with a reported location uncertainty > 5 km. We post‐processed the data (R package “CoordinateCleaner”; Zizka et al. 2019) to remove occurrences (1) with clearly erroneous coordinates (e.g., falling in the sea or with equal longitude and latitude) or (2) within 1 km from biodiversity institutions (botanical gardens, herbaria, universities, and museums), country centroids and GBIF headquarters. We also retrieved the native range reported for each species from the database of Plants of the World Online (POWO, https://powo.science.kew.org/, accessed 2022). We then removed species with less than 8 presences after resampling them at 1 km resolution in the native or invaded range, following the suggestion by Broennimann et al. (2012) of the minimum sample size required for niche shift analyses. After processing the GBIF data, we were left with 80 species, with a mean of 1861 presences in the invaded range (min = 34, max = 7700) and 1184 presences in the native range (min = 8, max = 22,300; Data S1).

We complemented the post‐processed presence database by sampling background environments for each species in both the native and the invaded range. We constrained the background sampled environmental conditions (which do not necessarily represent true species absences) within a buffer area of 100 km around each species' presence in the native and invaded range (in some cases extending beyond the biogeographical region of Mediterranean Europe). This approach aimed to capture the environmental conditions available for each species establishment, while ensuring a proper characterization of the shared environmental space across the native and invaded ranges (i.e., analogue environments). Constraining the environments reachable by the species to less than 100 km made little ecological sense, given that the alien species considered here have an average residence time of more than 150 years and are generally favored by human‐assisted dispersal, spreading as far as 5 km for each dispersal event over their residence time (Lososová et al. 2023, see Data S2.1 for some estimates of the dispersal distance for our species). In addition, comparing results with a 10 km buffer showed that restricting the sampled environments too much resulted in artificially constrained niche dimensions, leading to lower estimates of expansion and unfilling (Data S2.1). In contrast, a 50 km buffer or an even larger 1000 km buffer size did not significantly alter niche shift results compared to the 100 km buffer used here (Data S2.1).

To estimate the environmental conditions occupied by species in their native and invaded ranges, we selected five commonly used variables (e.g., Hageer et al. 2017) that jointly represent the relevant environmental conditions for the selected alien plant species. In the selection, we also included bulk density, a soil‐related variable that is often overlooked in similar analyses (Liu et al. 2020). As we focused on the analogue environments for our niche metrics (which always include the invaded range of the Mediterranean biogeographic region), we ensured that some of the selected variables were also particularly relevant in the seasonally dry and warm Mediterranean Basin (Cao Pinna et al. 2024, Deitch et al. 2017, see also the strong relation among the selected variables and overall environmental variability in Data S2.2). These selected variables were annual temperature range (bio‐07), mean temperature of the warmest quarter (bio‐10), precipitation of the wettest quarter (bio‐16) and precipitation of the driest quarter (bio‐17), which we obtained from the CHELSA database at approximately 1 km resolution (Karger et al. 2017). We included soil bulk density from the SoilGrids database (250 m resolution, Hengl et al. 2017), which is an excellent complement to precipitation since it captures soil water holding capacity and proved to be relevant for these species (Cao Pinna et al. 2024). We followed the suggestion of Bates and Bertelsmeier (2021) and limited the number of used environmental variables to control the size of the estimated niche and avoid identifying niche shifts in non‐relevant environments. To further explore our variable selection, we performed PCA to ensure that the chosen variables effectively represented the environmental variability for our presence points, while also exploring the covariance relations between climatic and soil variables separately (see Data S2.2). Finally, all environmental layers were resampled to 1 km resolution using bilinear interpolation, and species presences and background points were similarly resampled to avoid spatial mismatch between datasets.

### 2.2 Quantifying Niche Dynamics

For each of the 80 study species, we quantified niche shifts that occurred following their introduction in Mediterranean Europe. First, the background environmental variables (native and invaded ranges together) were summarized using a Principal Component Analysis (PCA) and then rescaled to an environmental grid (100 × 100 cells) using a kernel smoother to estimate densities (Broennimann et al. 2012, package “ecospat” version 3.2, Di Cola et al. 2017). This allowed us to directly compare the native and invaded niches using the first two axes of the PCA that captured 73% ± 4% of the total variance in the environmental space on average across species, while also partially controlling for sampling and environmental biases. We determined the overlap of the occurrence densities in the environmental space by estimating Schoener's *D* (Schoener 1970), which ranges between 0 (no overlap) and 1 (complete overlap). To test if the native niche is more similar than expected to the invaded one, we compared the observed *D* value with those obtained from 1000 random invasive niches (i.e., testing if the observed overlap is higher than 95% of simulated niches). For each species, we used the observed and random D scores to (1) determine if each species has shifted its niche or not (*p*‐value threshold of 0.05), and (2) quantify niche shift strength (measured as 1 − *D*).

We further decomposed niche dynamics into expansion and unfilling, focusing on the analogue environmental space. Niche expansion was measured as the percentage of the niche in the invaded range that is not occupied in the native range (and represents, along with stability, 100% of the invaded niche). Niche unfilling was measured as the percentage of the native niche not occupied in the invaded range. In subsequent qualitative explorations, we used a 10% threshold to distinguish minimal from significant expansion and unfilling, following the relevant literature (Petitpierre et al. 2012; Bates et al. 2020; Liu et al. 2020).

### 2.3 Identifying the Ecological Characteristics That Drive Niche Shifts

We tested whether: (1) residence time, (2) species dispersal ability, (3) biogeographic origin, (4) native environmental niche breadth, (5) functional traits, or (6) their variability (i.e., ITV or species plasticity) were good predictors of niche expansion and unfilling. We quantified residence time as the number of years since the first record in any of the countries of our study area, using the freely available database from Seebens et al. (2017). The maximum dispersal distance for each species was defined following the approach proposed by Tamme et al. (2014) using the R package “dispeRsal” using dispersal syndrome, growth form, seed mass, and plant height as predictors (all obtained from the TRY database; Kattge et al. 2020). As an indicator of biogeographic origin, we used the biome of origin from Cao Pinna et al. (2021) and aggregated species into three broad bio‐climatic categories: temperate, tropical, and Mediterranean/xeric. We defined the degree of native environmental niche breadth as the size of the multivariate environmental space occupied by each species in its native range using a minimum convex polygon (i.e., convex hull using the R package “adehabitatHR”). In terms of functional traits, we chose those that jointly describe a species' competitive ability: specific leaf area (SLA), plant height, seed mass, growth form (i.e., herbs, shrubs, trees), and life span (i.e., perennial/annual). SLA relates to the ability to capture resources rapidly (i.e., higher SLA values reflect faster growth rates), plant height captures the ability to compete for light in the adult stage, seed mass is related to growth rate and competitive ability in the seedling stage and to dispersal (Gioria et al. 2023), while growth form and life span provide a more integrated view of a species' competitive strategy. We obtained values for these traits from the TRY database, and for the three continuous traits, we calculated an average trait value for each species. To estimate the degree of ITV in these three traits, we calculated the coefficient of variation across all available records for each species (ITV was not related to sample size—Data S1 for each species data). We explored ITV since it potentially relates to both species genetic variability and plasticity, the major means through which plant populations can respond to various abiotic conditions (Karbstein et al. 2020). High ITV in SLA can affect photosynthetic efficiency and growth rates under different light and water availability (Karbstein et al. 2020), while seed mass can influence dispersal and germination abilities in different soil types (Kang et al. 2022; Gioria et al. 2023). Similarly, high ITV in plant height may reflect competitive ability for light in different light availability and disturbance settings (Siefert et al. 2015).

We used multiple regression models with a beta distribution (specific for percentages—using the R package “glmmTMB”) and an interaction term among residence time and every ITV to test the influence of these drivers on the percentages of (1) niche expansion and (2) unfilling. Stepwise variable selection based on Akaike's Information Criterion (AIC) was used to simplify the models. Moreover, given the complexity of the variable structure, we complemented the model selection process with a Random Forest (R package “randomForest”) and Multimodel Inference (R package “MuMIn”) to assess the consistency of variable selection between modelling choices (showing high agreement among model selection techniques, see Data S2.3). Given that closely related species may be non‐independent, we also tested for phylogenetic signals in our niche shift metrics. To do so, we first built a custom phylogeny for our species, based on the backbone phylogeny of vascular plants (Smith and Brown 2018), and then we calculated Blomberg's *K* (Blomberg et al. 2003) and Pagel's lambda (Pagel 1999) indices for our two niche shift metrics of unfilling and expansion (Data S2.4). As we found a lower phylogenetic signal than expected by chance for all metrics, we did not account for species phylogenetic relatedness in our models.

### 2.4 Linking Niche Shift to Invasion Success

We tested whether shifting the niche gave an advantage in different invasion success components, taking into account the confounding effect of residence time. To characterize the invasion success of each species in Mediterranean Europe, we used the results of Cao Pinna et al. (2021), who analyzed 130,000 vegetation plots from the European Vegetation Archive (EVA; Chytrý et al. 2016). From these, they calculated each species' commonness in terms of its: invaded range size (i.e., the geographic regional spread) and local abundance (i.e., the mean cover across plots where it occurs, see Cao Pinna et al. 2021 and Data S2.5 for a map of the distribution of the invaded plots used for the analysis). We used structural equation modeling (R package “piecewiseSEM”), and multiple linear regression analysis (except for niche shift—where we applied a binomial logistic regression model) to investigate the direct and indirect drivers of non‐native species' invaded range size and local abundance. Specifically, we tested (1) the effect of residence time on niche shift (binary variable consisting of two levels, “shifted” or “not shifted”) and on niche expansion; (2) the direct and indirect effects of residence time, niche shift, and expansion on the regional spread and local abundance (Figure 3). Even though niche shift comprises both expansion and unfilling, we specifically tested the role of niche expansion as it is generally associated with processes favoring invasion success, for example, local adaptations or enemy release. We did not test unfilling because of its high association with niche shift strength (Spearman's rank correlation rho = −0.75). The global goodness‐of‐fit of the SEM was evaluated via Fisher's *C* statistic (*p* > 0.05). Again, we ruled out the need to account for phylogeny in the analyses (Data S2.4). In Data S2.6, we repeated the same analysis, including the non‐analogue space in the calculation of niche dynamics (see Data S1 for the actual values), that is, non‐constraining the sample environments to the analogue space, but results remained largely unchanged.

## 3 Results

### 3.1 Is Niche Shift Common in Mediterranean Europe?

**FIGURE 1:**
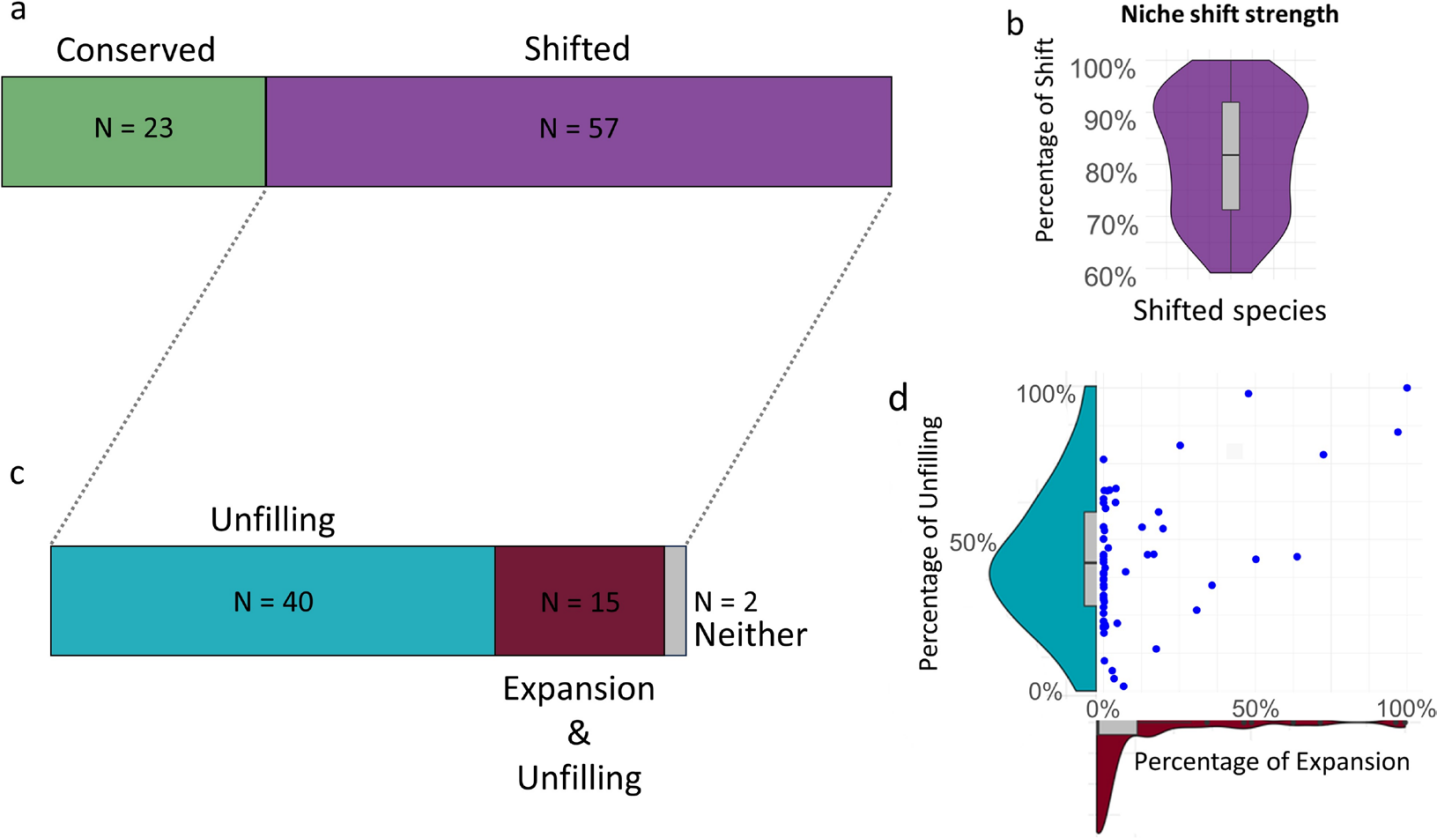
Bar plots showing the number of species that conserved or shifted their niche (a). Violin plot, with the niche shift strength for all niche shifting species identified in Mediterranean Europe (b). Barplots, showing the number of species that shifted their niche and had more than 10% expansion or unfilling (c, 2 species had neither more than 10% expansion nor unfilling in the shared environmental space—but still shifted their niches, for example, when they had little overlaps in the environmental space). Violin charts combined with a scatter plot show the strength of expansion and unfilling for the niche‐shifting species (d).

We observed that niche shifts were common across the 80 most widespread alien plant species in Mediterranean Europe, with 57 species (71%) occupying environments significantly different from those in their native range (Figure 1a,b for niche shift strength). These shifts were mainly due to unfilling (average = 42%; SD = 23%), with (40 + 15) out of 57 species (Figure 1c) experiencing at least 10% of unfilling. By contrast, niche expansion was much rarer (average = 11%; SD = 23%), with only 15 out of 57 niche‐shifting species showing at least 10% expansion (Figure 1c,d).

### 3.2 Which Ecological Characteristics Influence Niche Filling, and Which Drive Niche Expansion?

**FIGURE 2:**
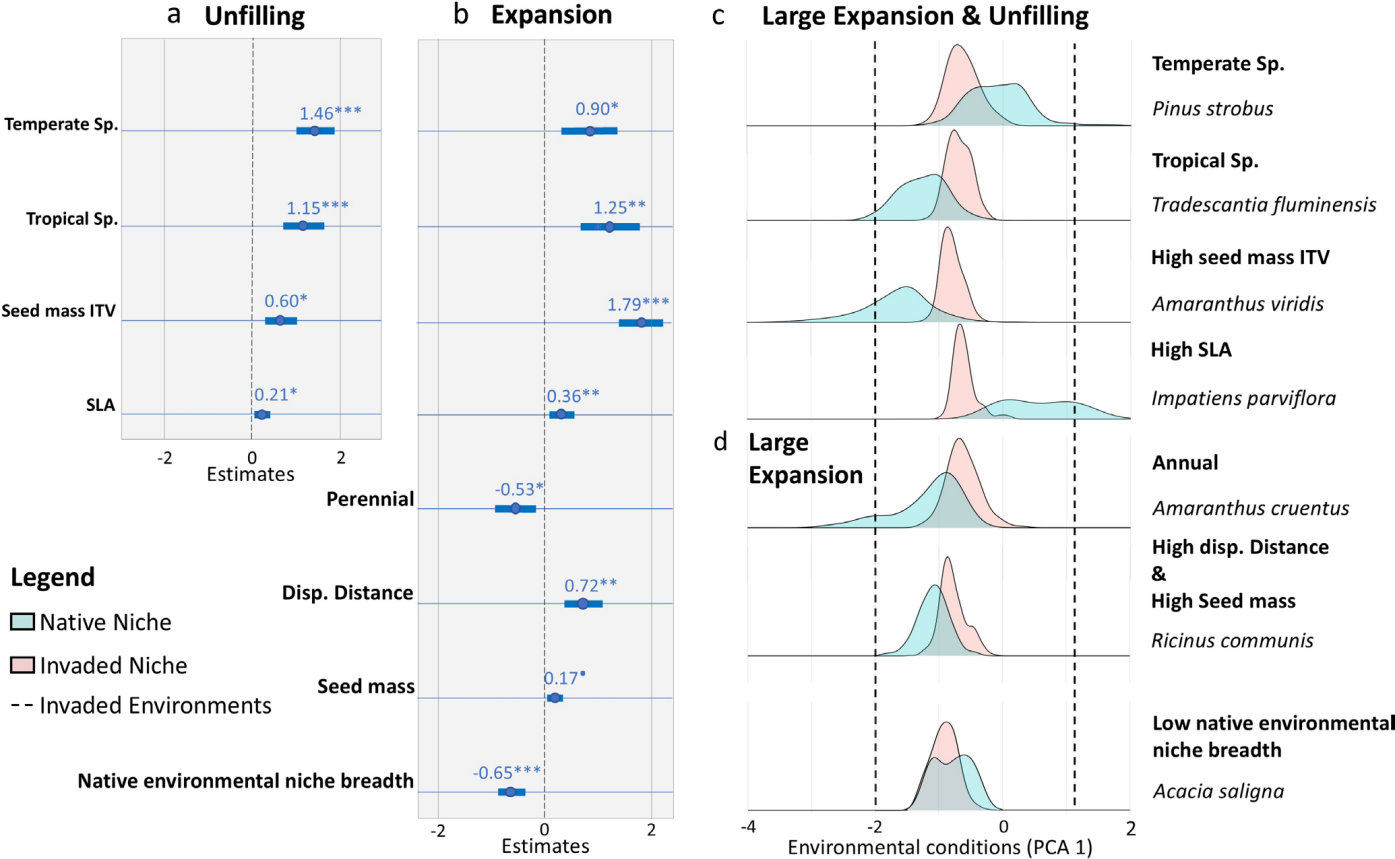
Importance of invader ecological features in niche dynamics. Standardized estimates (±Std. Error) for each significant predictor (after model selection) of the multiple beta regression for (a) the best model for explaining the level of niche unfilling and (b) the best model for explaining niche expansion. Blue bars represent a statistically significant relation (with an asterisk for the significance values). Estimated values for the categorical variables are compared to Mediterranean/xeric species for the biomes of origin, and compared to “annual” for the life span. (c and d) Illustration of individual species niche comparison between the native (blue) and invaded range (red), presented on the first axis of a PCA calibrated using the global environmental conditions (for better comparison among all species). Sampled species are illustrative examples of how species characteristics influence realized niche dynamics. ·*p* = 0.08, *p < 0.05, **p < 0.01 and ***p < 0.001.

Our best beta regression models for expansion and unfilling could explain 51% and 29% of the variance, respectively. Niche expansion was found to be higher in species with high dispersal distance, with high mean SLA and seed mass (only marginally significant), and high seed mass variability, in annual species, and those introduced from temperate and tropical biomes (*𝜙* = 5.5; *p* < 0.0001, *R* ^2^ = 0.51, d.f. = 10; Figure 2a,c for specific examples). In contrast, it significantly decreased with native niche breadth. We found that the degree of unfilling significantly increased with mean SLA, species trait variability (in seed mass) and for some biomes of origin (temperate and tropical, compared to the Mediterranean; *𝜙* = 5.2; *p* < 0.0001, *R* ^2^ = 0.29, d.f. = 6; Figure 2b–d for specific examples). Residence time was tested as a main effect or in interaction with ITV, as well as the other tested variables, such as plant height, growth form and ITV for some traits, although potentially relevant, were not significant for these final models. Furthermore, we tested global residence time—the time since the initial escape event—in a separate model and found it was non‐significant (see Data S1 for these additional residence time values).

### 3.3 Is Invasion Success in Mediterranean Europe More Frequent in Niche‐Shifting Species or in Species That Conserve Their Niche?

**FIGURE 3:**
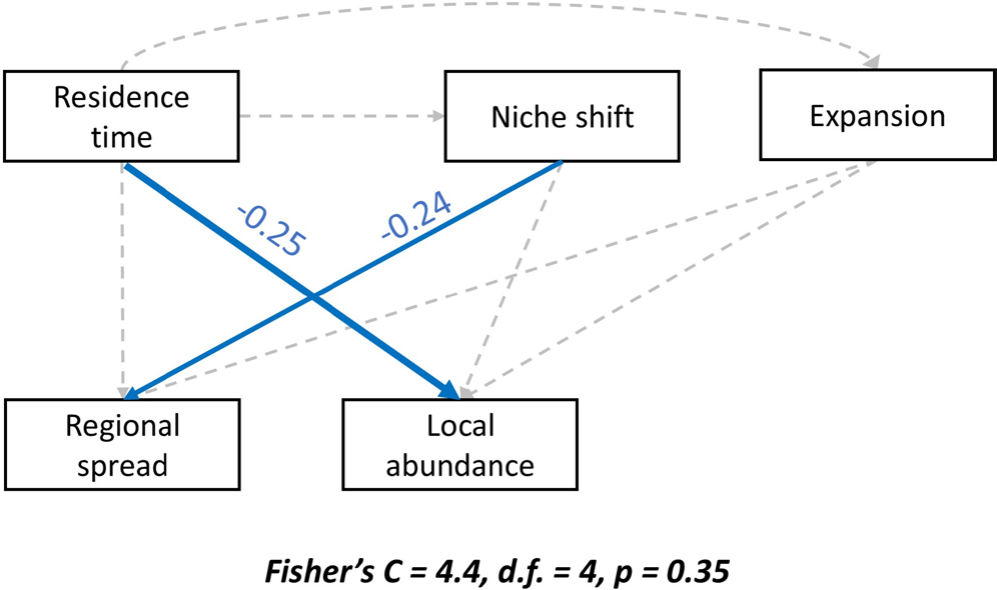
Results of the structural equation model used to test the direct single effect of residence time on niche shift (used as a binary variable: “shifted the niche” or “not shifted”) and expansion. The model was also used to test the combined effect of residence time, niche shift and expansion on the two facets of invasion success: local abundance and regional spread. Blue arrows represent significant relations (the arrow's size refers to the std. estimate intensity, i.e., the value on the arrows), and grey dashed lines refer to the tested but not significant relations.

Our structural equation model (Fisher's *C* = 4.4, d.f. = 4, *p* = 0.35) showed only the direct effects of residence time and niche shift on invasion success, measured as the regional spread and local abundance, but not an interplay between them or niche expansion. We found that a species' ability to conserve its native niche, rather than shifting it, favored invasion success in Mediterranean Europe (Figure 3). Specifically, we found that residence time had no effect on niche shift, and species that conserved their niches (“not shifted” as a binary output) were more regionally widespread in the invaded range (std. estimate = −0.24; df = 76; *p* = 0.03; Figure 3). Conversely, niche shifting or conservatism did not significantly influence local abundance in the invaded range (std. estimate = −0.08; df = 76; *p* = 0.47; Figure 3) while residence time did (std. estimate = −0.25; df = 76; p = 0.03; Figure 3).

## 4 Discussion

### 4.1 Realized Niche Shifts Are Common in Mediterranean Europe

Our results show that most of the alien plants introduced in Mediterranean Europe occupy environmental conditions that are different from those in their native range, suggesting that realized niche shifts are common in this area. Most of these species shifted their niche primarily because of their inability to fill the environmental conditions already occupied in their native range (i.e., unfilling) rather than because of niche expansion (Atwater et al. 2018; Bates et al. 2020; MacLean and Beissinger 2017; Strubbe et al. 2013), although the two values were slightly correlated (Figure 1d).

Compared to earlier studies on alien plants, our results show more frequent niche unfilling and less frequent niche expansion (e.g., Sychrová et al. 2022 for introduced trees and shrubs, González‐Moreno et al. 2015 for introduced *Oxalis*). These differences with other studies may be due in part to methodological choices and differences between study systems. First, most studies have used only climatic variables to characterize species niches, while we used climatic and edaphic information. This increase in the number of dimensions of the niche space makes the niche estimation more realistic, but also statistically more likely to observe unfilling patterns (e.g., when species are constrained by unfavourable edaphic conditions). Second, our methodological choice to select only analogue environments may have forced differences with other works (e.g., Liu et al. 2020). In theory, this choice could affect the niche dynamics (Bates and Bertelsmeier 2021), but in our case, it did not significantly change the main trends (see re‐analyses with non‐analogue conditions in Data S1 and S2.6). Third, compared to previous studies, we also analyzed herbaceous growth forms, including therophyte species. Our focus on rather short‐lived species that spend part of their life cycle as seeds and have different tolerance to stress may influence their uses and transport by humans (Atwater et al. 2018), as well as establishment and colonization rates. Indeed, a study on *Acacia longifolia* (Andrews) Willd introduction to Mediterranean climates (Dinis et al. 2020) found similar niche dynamics to those we observed in our *Acacia* species. These peculiar niche dynamics may also be linked to the harsh seasonally dry environmental conditions of the Mediterranean climate. In fact, we found that pre‐adapted Mediterranean invaders tend to have less niche unfilling than species originating from other biomes. This may reflect the presence of unmeasured specificities of the Mediterranean region, which can be either linked to climate (e.g., influencing fire regimes) or biotic (biotic resistance due to generally high community richness), limiting the ability of the non‐pre‐adapted alien species to fill their native niche and to expand in novel environments.

### 4.2 Niche Dynamics Are Explained by Dispersal Limitations and Biotic Interactions, While Acclimatization and Local Adaptation May Also Play a Role

Like several previous authors, we found that residence time did not influence species niche dynamics (Dellinger et al. 2016; Early and Sax 2014; Petitpierre et al. 2012). In a region with long human exploitation, such as Mediterranean Europe, processes more complex than just time for dispersal may be at play, and species ecological characteristics were better predictors of both niche unfilling and niche expansion.

For example, we found that the intrinsic dispersal ability of the species favors niche expansion. One potential explanation is that many alien species were limited in their native range by extrinsic geographic barriers, such as mountain ranges or water bodies, that could not be overcome naturally (as theorized, e.g., by MacLean and Beissinger 2017), irrespective of their dispersal ability. Only in the invaded range and thanks to the human‐mediated introductions, their natural dispersal ability becomes an important factor favoring expansion in novel climates (Bates et al. 2020; Menuz et al. 2015; Smith et al. 2020). This interpretation is also congruent with the finding that species with broad native environmental niche tolerance have minimal expansion since they are typically not dispersal‐limited in their native range (Bates and Bertelsmeier 2021; Early and Sax 2014). Moreover, this result is in line with other studies (e.g., Bates et al. 2020; Dellinger et al. 2016) finding that species with a narrow native niche tend to have more expansion because they could benefit more from human‐mediated dispersal, allowing them to access novel climatic conditions within their fundamental niche.

Our results are also consistent with the idea that niche shift dynamics are influenced by the novel biotic interactions established in the invaded range, supporting the importance of enemy release and maintaining positive mutualistic interactions. We found that species with large seeds, which are theoretically poor competitors at the seedling stage, slow growers, and poor invaders (as they generally also have small seed production; see e.g., Gioria et al. 2023), were more likely to expand their niche after being released from their native range competitors and enemies (Brian and Catford 2023). We also found that species with high SLA tended to expand their niche, despite being fast in resource acquisition and hence competitive species in disturbed environments, which are theoretically not strongly limited at home. However, these fast‐growing species are likely the ones with the least investment in defenses from enemies (de Vries et al. 2019; Brian and Catford 2023). Thus, once freed from pathogens, herbivores, and competitors of their native range, their high competitive ability may favor the expansion of their niche (Mitchell and Power 2003; Brian and Catford 2023). Another interesting novel result is that climatically pre‐adapted species from other Mediterranean/xeric biomes have less expansion and unfilling in the invaded range than temperate and tropical species (see examples of *Pinus strobus* L. and *Tradescantia fluminensis* Vell., respectively, in Figure 2). This might seem like an obvious finding, given that Mediterranean climates are more likely to be similar, and we might intuitively expect the probability of observing a niche shift to be lower. However, since we restricted our main analysis to the analogue environments, comparing two very similar Mediterranean climates may force a broader overlap in the analogue environmental space compared to the other biomes, but not necessarily lead to less expansion or unfilling. Actually, a greater available analogue environment should instead allow detecting more unfilling, which is the opposite of what we find. Our results thus support the idea that Mediterranean species are indeed well pre‐adapted and are thus less likely to shift their niche. In addition, species of Mediterranean/xeric origin may find ecologically similar competitors and herbivores (“analogue” enemies) in the invaded range, resulting in limited enemy release and less expansion (e.g., according to the framework proposed by Brian and Catford 2023). Similarly, these climatically pre‐adapted Mediterranean/xeric species like our *Acacia saligna* (Labill.) H.L.Wendl., which comes from comparable environmental conditions, may find mutualist species in the invaded region (Mucina 2019; Olson et al. 2001). This may allow them to conserve some of the positive ecological interactions already established in their native range, favoring niche filling if compared with temperate and tropical species, as we found in our analysis.

Finally, we found that species with greater ITV in some traits (specifically in seed mass; Violle et al. 2009; Kang et al. 2022) were subject to more niche expansion. This result aligns with our expectations that species that are either plastic or that host high genetic diversity (both features generally correlated with high ITV) are better able to establish in new environmental conditions, compared to species with low plasticity or genetic diversity (Smith et al. 2020; Kang et al. 2022). The finding that species with more variable seeds may be able to expand better in novel environments suggests this may be related to their greater acclimatization (Davidson et al. 2011; Kang et al. 2022) or even adaptive potential, which may be important in the early stages of invasion. Indeed, it has been shown that invading plants can show acclimatization (e.g., Bujan et al. 2021) or quickly adapt during range expansion (Colautti and Barrett 2013). Species with more variable seeds (rather than with more variable SLA or height) may be advantaged in a wider range of environments because of their higher persistence in the soil seed bank and establishment ability (Gioria et al. 2023, 2021; Kang et al. 2022). While a high ITV can be an indicator of a high adaptive potential that favors expansion, it was also associated with greater unfilling. This result suggests that the niche of the species with greater ITV may be less stable in the invaded range, potentially needing a longer time to reach stability than the niche of species with lower ITV.

Nevertheless, we recognize that we measured ITV at the global scale, and we do not know the actual genetic variability or phenotypic plasticity introduced into the invaded range. We also do not know if global ITV reflects the variability in each species' native range. In this complex framework, data limitations forced us to consider ITV not only as a proxy for a single mechanism, but rather as a composite signal of both genetic diversity and plasticity. However, this is the first study finding a link between ITV and niche shifting, suggesting that further studies are needed to address experimentally whether shifts are related to plasticity or may even have a genetic basis (Bates and Bertelsmeier 2021), providing evidence for adaptive evolution as a driver of a niche shift. Therefore, the interpretations of this specific result should be carefully evaluated and further tested in future research (e.g., whether the niche expansion was enabled by genetic changes, plasticity or something else), also given the fact that of the three traits tested, only seed mass showed a significant trend for ITV.

### 4.3 Niche Conservatism in the Invaded Range Explains Invasion Success

Contrary to the expectation that rapid local adaptation or acclimatization is one of the reasons why alien species become invasive (e.g., Bujan et al. 2021; Colautti and Barrett 2013; Sotka et al. 2018; Lancaster et al. 2015), our results suggest that invasion success, measured as the regional spread, is not driven by species' capacity to expand their niche, but rather to conserve it (Bates et al. 2020; Liu et al. 2020; Petitpierre et al. 2012). We should acknowledge that species that are more widespread in the invaded area are more likely to have reached all available environmental conditions, so it is somewhat expected that they would show less unfilling. However, we note that our measure of regional spread was assessed with a completely independent dataset from the one used to quantify unfilling. In addition, similar to our results, a recent study on ants (Bates et al. 2020) showed that the propensity to shift the niche is also not related to the species' ability to cause impacts or spread more broadly. The most plausible explanation is that species spread more successfully under environmental conditions they have encountered in their native range and to which they are preadapted (Sychrová et al. 2022). This advantage is maximized when they can rely on human‐mediated dispersal or are released from negative biotic interactions, such as competition or predation. Biotic interactions can also influence the dynamics of the acute phase of invasion. Our results show that during the early stages of invasion, species tend to have higher local abundance, potentially causing more impacts, similar to the findings of Cascone et al. (2021). This aligns with the explanation by Brian and Catford (2023), which proposed that alien species initially benefit from being released from competitors and predators in their invaded range. However, as aliens' abundance grows, generalist predators in the invaded range may begin to target them, limiting their expansion and abundance (Levine et al. 2004).

Overall, our results allow us to pinpoint the main processes shaping the dynamics of niche shifts in invasive species. Despite the long history of plant invasions in Mediterranean Europe, many invasive species have not yet filled the environmental conditions analogous to their native niche. Indeed, the residence time of alien species has little effect on the niche shift dynamics in Mediterranean Europe, but it has an important impact on invasion success, suggesting the need for further studies exploring this relation. Specifically, we identified dispersal limitations, biotic interactions, and acclimatization or even local adaptations as the main processes influencing niche dynamics during invasions. In addition, we found that the ability of the species to conserve their niche and to establish in the environmental conditions to which they are already pre‐adapted (niche filling) is what favors their invasion success most.

## Author Contributions

**Luigi Cao Pinna:** conceptualization, data curation, formal analysis, funding acquisition, investigation, methodology, resources, software, supervision, validation, visualization, writing – original draft, writing – review and editing. **Laure Gallien:** conceptualization, investigation, methodology, validation, visualization, writing – original draft, writing – review and editing. **Tommaso Jucker:** conceptualization, formal analysis, investigation, methodology, supervision, writing – original draft, writing – review and editing. **Milan Chytrý:** conceptualization, data curation, investigation, resources, supervision, writing – review and editing. **Greta La Bella:** formal analysis, methodology. **Alicia T. R. Acosta:** data curation, investigation, resources, supervision, validation, writing – original draft, writing – review and editing. **Marta Carboni:** conceptualization, formal analysis, funding acquisition, investigation, methodology, project administration, supervision, validation, visualization, writing – original draft, writing – review and editing.

## Conflicts of Interest

The authors declare no conflicts of interest.

## Supporting information


**Data S1:** gcb70379‐sup‐0001‐DataS1.xlsx.

**Data S2.** gcb70379‐sup‐0002‐DataS2.pdf.

## Data Availability

The data and code that support the findings of this study are openly available in Dryad at https://doi.org/10.5061/dryad.cc2fqz6gc. Plant occurrence records were obtained from GBIF at https://doi.org/10.15468/dl.mdkwsz. The native ranges for each species were identified using Plants of the World Online (POWO), available at https://powo.science.kew.org/. Environmental data of present‐day climate were obtained from CHELSA at https://chelsa‐climate.org (Version 1.2). Soil bulk density data were obtained from ISRIC at https://data.isric.org/geonetwork/srv/eng/catalog.search#/metadata/2ebc3811‐2783‐4fb6‐bb68‐e8af47d32fd3 (Version 1.0). The traits data were obtained from TRY at https://www.try‐db.org/ (Version 5.0).
