# Supplementary material for: Niche Dynamics of Alien Plant Species in Mediterranean Europe
Source: Glob Chang Biol. 2025 Aug 1;31(8):e70379. doi: 10.1111/gcb.70379 (PMC12315792; doi:10.1111/gcb.70379)
Supplement: Supplementary file 2 — Data S2. gcb70379‐sup‐0002‐DataS2.pdf. [file GCB-31-e70379-s001.pdf]

Supporting information for the manuscript: ”Niche dynamics of alien plant species in Mediterranean Europe”

S2.1 Sensitivity on the buffer

The range of sampled environments can marginally influence niche shift analyses. That’s why we complemented the 100 km buffer (as in the main text) around each species’ presence in the native and invaded range, with different buffer values (i.e., 10 and 1000 km). Selecting the appropriate sampling area presents a two-fold challenge. On the one hand, using a too-restrictive buffer may limit the dimensions of the analogue environment, artificially constraining niche expansion and unfilling - like how niche truncation affects species distribution models. On the other hand, sampling an excessively broad area can include analogous environments that are not reachable for the species, but more importantly, problematic for niche similarity tests. As noted by Bates & Bertelsmeier (2021), an overly large environmental sample can lead to a systematically low expected overlap under the null hypothesis (i.e., of a random shift of the niche in the shared environments), artificially inflating the significance of niche shifts. That’s why we analysed if the niche shift dynamics differ significantly from our 100 km buffer, testing the couples using a paired t-test. We found no differences in results considering the bigger 1000 km buffer. This result may indicate that, in our case (where the invaded range is geographically constrained by the sea), oversampling does not pose a methodological concern. No differences were found for the 50 km buffer, again confirming the validity of the selected buffer we presented in the main results. For the smaller buffers, we found some differences. Significant differences were found for the 10 km buffer (i.e., lower mean expansion, unfilling and more D overlap), confirming that by limiting the sampling of background values, we are artificially constraining the dimension of the shared environmental space (i.e., niche shift truncation).

Regarding the ecological reasons for the chosen buffer, we followed a recent work (Lososová et al., 2023), according to which alien species spread by anthropochory (i.e., human-assisted dispersal) may allow seeds to disperse as far as 5 km for each dispersal event. This is also confirmed by our estimations of a mean dispersal distance of 2.5 km for our set of 80 alien species (Supporting S1). Indeed, we estimated the reachable environments by multiplying each species' residence time by the maximum dispersal estimate. In these cases of human-assisted dispersal, we may expect our alien species to have an average maximum dispersal distance of as far as 781 km (using 5 km as the dispersal estimate found in the literature) or 335 km (for our species-specific dispersal distance estimate). Anyway, we appreciate that a buffer larger than 300 km is unrealistic and not needed, given we may be overestimating the dispersal distance, but regardless, bigger buffers do not change our main trends.

Table S2.1 Sensitivity on the buffer. The paired t-test comparison was performed among different buffer dimensions used for background environment sampling. To correct for multiple tests comparison, we applied a Bonferroni correction (0.05/12 = 0.004), which was then used to identify significant patterns (shown in red).

	**Niche overlap**	**p-value**	**Expansion**	**Unfilling**
**100 vs 10 km**	$t = -4.3$<br>$df = 79$<br>p-value = $4.1 * 10^{-5}$	$t = 4.9$<br>$df = 79$<br>p-value = $5.2 * 10^{-6}$	$t = 2.18$<br>$df = 79$<br>p-value = 0.032	$t = 8.3$<br>$df = 79$<br>p-value = $2.4 * 10^{-12}$
**100 vs 50 km**	$t = -0.060$<br>$df = 79$<br>p-value = 0.95	$t = 2.55$<br>$df = 79$<br>p-value = 0.013	$t = 0.64$<br>$df = 79$<br>p-value = 0.52	$t = 2.26$<br>$df = 79$<br>p-value = 0.027
**100 vs 1000 km**	$t = -0.3$<br>$df = 79$<br>p-value = 0.79	$t = -0.28$<br>$df = 79$<br>p-value = 0.78	$t = 0.21$<br>$df = 79$<br>p-value = 0.83	$t = -2.1$<br>$df = 79$<br>p-value = 0.04

S2.2 PCA for the environmental variables

To explore the covariance relation among our selected variables and all those available in CHELSA and SoilGrid we performed two PCAs. These analyses used all global alien species' presences to explore the importance of our selected variables in the general environmental trends, and to define relations among the chosen predictors and all the others. The PCA in Figure S2.2a shows that the climatic variables we selected (red arrows) strongly relate to the other CHELSA ones (see the figure legend for an explanation of the name codes). The selected climatic variables also strongly relate with the first two axes of the PCA (PC1 = bio-07 = 58%; bio-10 = -58%; bio-16 = -57%; bio-17 = 21%; PC2 = bio-07 = 34%; bio-10 = 39%; bio-16 = -58%; bio-17 = -82%). The PCA in Figure S2.2b shows that Bulk density, our selected soil variable, is the most strongly related to the first axis of the PCA (97 %) and is capturing most of the covariance of the other soil variables (but less of those related to the second PCA axis).

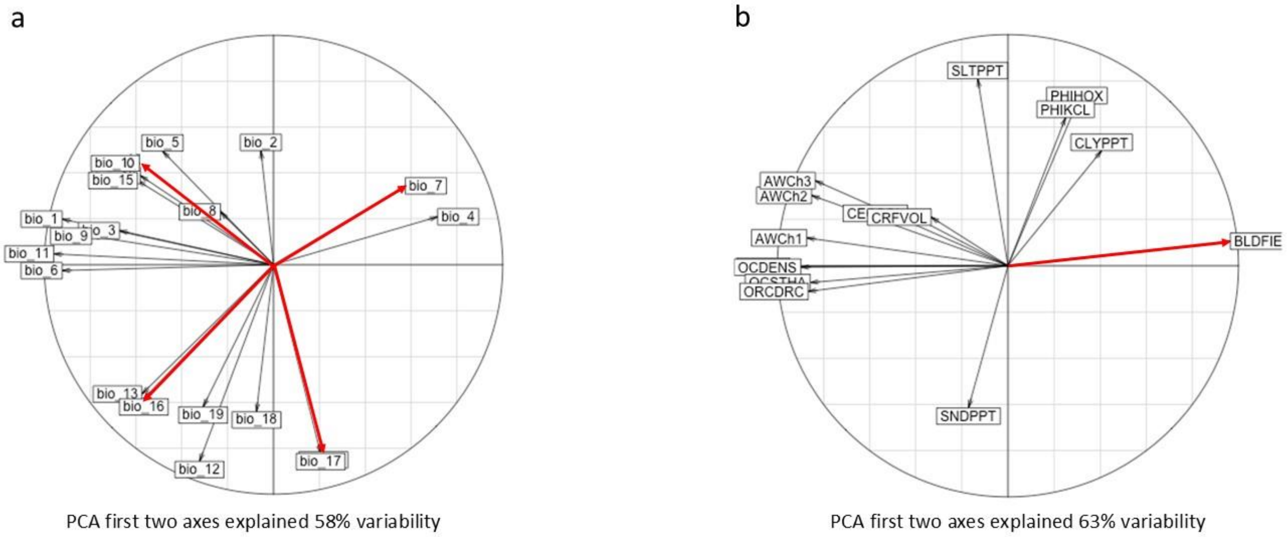

Figure S2.2: PCAs exploring the covariance for the climatic (a, bio-01 = Annual Mean Temperature, bio-02 = Mean Diurnal Range (Mean of monthly (max temp - min temp)), bio-03 = Isothermality (BIO2/BIO7) (×100), bio-04 = Temperature Seasonality (standard deviation ×100), bio-05 = Max Temperature of Warmest Month, bio-06 = Min Temperature of Coldest Month, bio-07 = Temperature Annual Range (BIO5-BIO6), bio-08 = Mean Temperature of Wettest Quarter, bio-09 = Mean Temperature of Driest Quarter, bio-10 = Mean Temperature of Warmest Quarter, bio-11 = Mean Temperature of Coldest Quarter, bio-12 = Annual Precipitation, bio-13 = Precipitation of Wettest Month, bio-14 = Precipitation of Driest Month, bio-15 = Precipitation Seasonality (Coefficient of Variation), bio-16 = Precipitation of Wettest Quarter, bio-17 = Precipitation of Driest Quarter, bio-18 = Precipitation of Warmest Quarter, bio-19 = Precipitation of Coldest Quarter) and soil from SoilGrid (b, AWCh1 = Percentage of Available soil water capacity (volumetric fraction)

with FC = pF 2.0, AWCh2 = percentage of Available soil water capacity (volumetric fraction) with FC = pF 2.3, AWCh3 = Percentage of Available soil water capacity (volumetric fraction) with FC = pF 2.5, , BLDFIE = Bulk density (fine earth, kg/m3), CECSOL = Cation Exchange Capacity of soil (cmolc/kg), CLYPPT = Weight percentage of the clay particles (<0.0002 mm), CRFVOL = Volumetric percentage of coarse fragments (>2 mm), OCDENS = Soil organic carbon density (kg/m3), OCSTHA = Soil organic carbon stock (ton/ha), ORCDRC = Soil organic carbon content (permille), PHIHOX = pH index measured in water solution, PHIKCL = pH index measured in KCl solution, SLTPPT = Weight percentage of the silt particles (0.0002–0.05 mm), SNDPPT = Weight percentage of the sand particles (0.05–2 mm)) variables extracted from the selected alien species global presences. In the first PCA using all CHELSA environmental variables, the first two axes explained 58% of the variability (a). For the PCA in panel b, the first two axes explained 63% of the total variability, showing the covariance relation among our selected variable of Bulk density and all the other available variables in SoilGrid. We used red arrows; to highlight the variables we used in the main text.

S2.3 Model and Variable Selection

Given the high number of variables in the model selection process, we explore the model selection procedure with a Random Forest (R package "randomForest") and Multimodel Inference (R package "MuMIn"). We assessed the consistency of the variable selection across models and how different modelling algorithms lead to similar model definitions and results. Multimodel inference is based on the assumption of multiple fitting (2^N, N= number of variables), of random models that could contain or not sequentially each variable and then ranking them based on specific criteria (AIC in our case). It is not surprising that the stepwise and the multimodal inference approaches completely agree with our set of predictors, as these approaches are both based on information criteria. However, it is partially surprising (and proving our model is robust) that the random forest approach, which is based on multiple trees and their aggregation (ensemble learning), also agrees with the final model selection (except for plant height) and predictions.

Table S2.3 Model and variable selection

	Selected Variables	Stepwise Model Selection (AIC)	Multi-Model Inference	Random Forest
Residence Time				
Life Cycle	x	x	x	
Life Forms				
Dispersal Distance	x	x	x	x
Biogeographic Origin	x x	x x	x x	x
SLA	x x	x x	x x	x x
Planth Height				x x
Seed Mass	x	x	x	x
SLA_ITV				
Planth Height_ITV				
Seed Mass_ITV	x x	x x	x x	x x
Generalism	x	x	x	x

x Expansion

x Unfilling

S2.4 No phylogenetic signal in response variables

We constructed a phylogenetic tree including all examined species using the “phylo.maker” function (“V.PhyloMaker” package; Jin & Qian, 2019) based on the backbone phylogeny of vascular plants from Smith & Brown (2018). Thus, we tested for the presence of a phylogenetic signal using two of the most common estimators, Blomberg’s K (Blomberg et al., 2003) and Pagel’s lambda (Pagel, 1999). Values of Blomberg’s K > 1 indicate higher levels of phylogenetic signal than expected, while K < 1 indicates less phylogenetic signal than expected. Similarly, Pagel’s λ ranges between 0 (lack of phylogenetic conservatism) and 1 (higher phylogenetic conservatism). Phylogenetic signals were estimated using the function “phylosig” in the “phytools” package (Revell, 2012). Results were consistent between the two methods.

Table S2.4 No phylogenetic signal in response variables

Response	Lambda	p-value	K	p-value
Obs_D_0	0.286566	0.274363	0.00775614	0.656
expansion_0	6.61164e-05	1	0.0639098	0.062
sim.test_D_p.value_0	6.61164e-05	1	0.0189328	0.285
unfilling_0	0.476212	0.141313	0.0195983	0.204
Mean_cover.y	0.161025	0.629734	0.00764535	0.756
PC1_range	6.61164e-05	1	0.0207869	0.145
longlatext	0.252916	0.468823	0.0326569	0.029

S2.5 Distribution of the vegetation plots used to evaluate invasion success

To evaluate the two components of invasion success in the invaded range, Cao Pinna et al., (2021) analysed 130,000 vegetation plots from the European Vegetation Archive (EVA; Chytrý et al., 2016). Here we showed the geographic distribution of the plots (invaded and not) used to evaluate these invasion success metrics in Mediterranean Europe. In the map, we have shown all the used plots to define the sampling effort of the dataset, while only the alien species presences were used to evaluate invasion success metrics i.e., regional spread and local abundance in our study area.

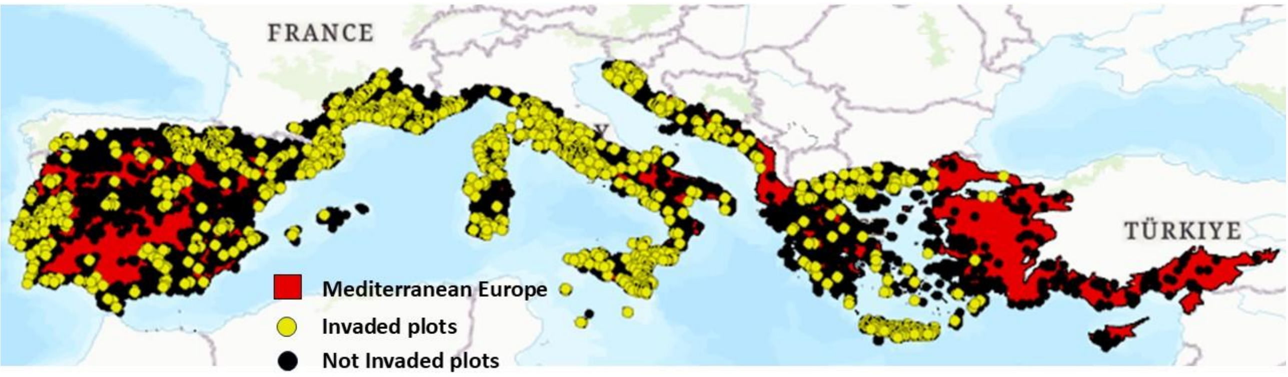

Figure S2.5: Map showing the geographic distribution of the plots used to evaluate invasion success metrics in our main text. Mediterranean Europe, our study area, is shown in red, and the location of the analysed invaded plots from the European Vegetation Archive (EVA) is represented in yellow. The invaded plots overlay on top of the non-invaded ones (in black).

S2.6 Invasion success in non-analogue space

To explore if the results we found regarding invasion success remain constant when choosing different niche shift metrics (which relate to different theoretical frameworks), we analysed how our results may change when considering the non-analogue environments (not limited to the analogue environments shared by the native and invaded niche). Indeed, we performed the same analysis presented in the main text but using the niche shift metrics estimated also considering the non-analogue environments (which showed a correlation test significance lower than 0.001 for all niche dynamics metrics and a correlation coefficient of 0.93 for expansion and 0.92 for unfilling, see S1 legend). These metrics are different from those presented in the main text, since accounting only for the analogue climates may cut out areas of expansion or unfilling that are in these non-analogue climates (generally called pioneering and abandonment), causing the identification of more niche stability (i.e., less expansion and less unfilling). Nevertheless, considering non-analogue environments may have unexpected consequences on the niche stability test (increasing or reducing the probability of identifying a niche shift, i.e., influencing our niche shift metric), since the test is randomly shifting the niche in a bigger environmental space, the dimension of which will depend on the number of background points selected and also on the overall sampled environmental space. Ultimately, this analysis reveals that regardless of the choice of including the analogue environments or not, our results do not change. This reinforces the reliability of our analysis and the consistency of our results.

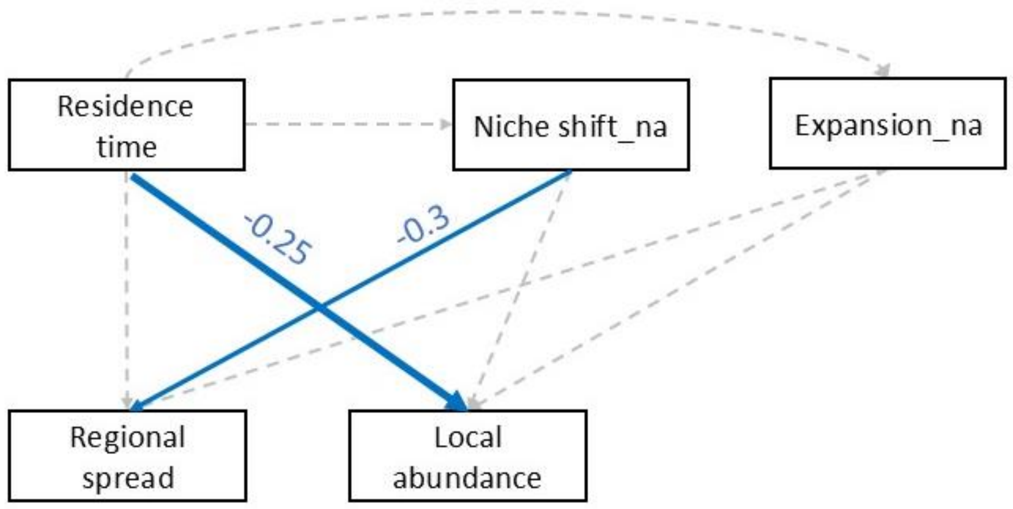

Fisher's C = 5.1, d.f. = 4, p = 0.28

Figure S2.6: Results of the structural equation model used to test the direct single effect of residence time on niche shift (used as a binary variable: “shifted the niche” or “not shifted”) and expansion, both evaluated in the non-analogue space (na). The model was also used to test the combined effect of residence time, niche shift_na (non-analogue) and expansion_na (non-analogue) on the two facets of invasion success: local abundance and regional spread (evaluated as in the main text). Blue arrows represent significant relations (the arrow’s size refers to the std. estimate intensity, i.e. the value on the arrows), and grey dashed lines refer to the tested but not significant relations. These results are not different from those of the main text.

References

Bates, O. K., & Bertelsmeier, C. (2021). Climatic niche shifts in introduced species. *Current Biology*, *31*(19), R1252–R1266. https://doi.org/10.1016/j.cub.2021.08.035

Blomberg, S. P., Garland Jr, T., & Ives, A. R. (2003). Testing for phylogenetic signal in comparative data: behavioral traits are more labile. Evolution, 57(4), 717-745. https://doi.org/10.1111/j.0014-3820.2003.tb00285.x

Cao Pinna, L., Axmanová, I., Chytrý, M., Malavasi, M., Acosta, A. T. R., Giulio, S., … Carboni, M. (2021). The biogeography of alien plant invasions in the Mediterranean Basin. *Journal of Vegetation Science*, *32*(2). https://doi.org/10.1111/jvs.12980

Chytrý, M., Hennekens, S. M., Jiménez-Alfaro, B., Knollová, I., Dengler, J., Jansen, F., … Yamalov, S. (2016). European Vegetation Archive (EVA): An integrated database of European vegetation plots. Applied Vegetation Science, 19(1), 173–180. https://doi.org/10.1111/avsc.12191

Jin, Y. and Qian, H. (2019), V.PhyloMaker: an R package that can generate very large phylogenies for vascular plants. Ecography, 42: 1353-1359. https://doi.org/10.1111/ecog.04434

Pagel, M. (1999). Inferring the historical patterns of biological evolution. Nature, *401*(6756), 877-884. https://doi.org/10.1038/44766

Lososová, Z., Axmanová, I., M. Chytrý, M., et al. (2023). Seed Dispersal Distance Classes and Dispersal Modes for the European Flora. *Global Ecology and Biogeography*, 34(3), 1485-1494. https://doi.org/10.1111/geb.70026

Revell, L. J., (2012). phytools: An R package for phylogenetic comparative biology (and other things). Methods Ecology and Evolution 3, 217–223. https://doi.org/10.1111/j.2041-210X.2011.00169.x

Smith, S. A., and Brown, J. W.. 2018. Constructing a broadly inclusive seed plant phylogeny. American Journal of Botany, 105(3): 302– 314. https://doi.org/10.1002/ajb2.1019
